# Effect of Long-Term Dietary Arginyl-Fructose (AF) on Hyperglycemia and HbA1c in Diabetic *db*/*db* Mice

**DOI:** 10.3390/ijms15058352

**Published:** 2014-05-12

**Authors:** Kwang-Hyoung Lee, Kyoung-Soo Ha, Sung-Hoon Jo, Chong M. Lee, Young-Cheul Kim, Kwang-Hoe Chung, Young-In Kwon

**Affiliations:** 1 Thrombosis and Vascular Biochemistry Lab., Department of Applied Science, CHA University, Gyeonggi-do 463-836, Korea; E-Mail: lkh1@chamc.co.kr; 2 Department of Food and Nutrition, Hannam University, Daejeon 305-811, Korea; E-Mails: kengkoo@nate.com (K.-S.H.); sunghoon04@hanmail.net (S.-H.J.); 3 Department of Nutrition and Food Science, University of Rhode Island, Kingston, RI 02881, USA; E-Mail: chonglee@mail.uri.edu; 4 Department of Nutrition, University of Massachusetts, Amherst, MA 01003, USA; E-Mail: yckim@nutrition.umass.edu

**Keywords:** type 2 diabetes, pre-diabetes, blood glucose, α-glucosidase inhibition, arginyl-fructose (AF)

## Abstract

We have previously reported that Amadori compounds exert anti-diabetic effects by lowering sucrose-induced hyperglycemia in normal Sprague-Dawley rats. In the present study we extended our recent findings to evaluate whether α-glucosidase inhibitor arginyl-fructose (AF) lowers blood glucose level in diabetic *db*/*db* mice, a genetic model for type 2 diabetes. The *db*/*db* mice were randomly assigned to high-carbohydrate diets (66.1% corn starch) with and without AF (4% in the diet) for 6 weeks. Changes in body weight, blood glucose level, and food intake were measured daily for 42 days. Dietary supplementation of AF resulted in a significant decrease of blood glucose level (*p* < 0.001) and body weight (*p* < 0.001). The level of HbA1c, a better indicator of plasma glucose concentration over prolonged periods of time, was also significantly decreased for 6-week period (*p* < 0.001). Dietary treatment of acarbose^®^ (0.04% in diet), a positive control, also significantly alleviated the level of blood glucose, HbA1c, and body weight. These results indicate that AF Maillard reaction product improves postprandial hyperglycemia by suppressing glucose absorption as well as decreasing HbA1c level.

## 1. Introduction

Non-insulin dependent diabetes mellitus (NIDDM, type 2 diabetes) is a common disorder of glucose and fat metabolism that affects approximately 171 million people worldwide, generating immense health care costs [1]. Type 2 diabetes accounts for about 90% to 95% of all diagnosed cases of diabetes in adults [2]. Pre-diabetes is a condition in which individuals have blood glucose levels higher than normal but not high enough to be classified as diabetes [3]. In the United States, in 2010, 25.8 million people (10% of American adults) had diabetes and by 2050 this figure is expected to jump to 33%, or one-third of all American adults [2].

Hyperglycemia is a condition characterized by a rapid rise in blood glucose levels, which is due, primarily, to increased hydrolysis of starch by pancreatic α-amylase and α-glucosidases, leading to enhanced absorption of glucose in the small intestine. One of the therapeutic approaches for decreasing postprandial hyperglycemia is thus to retard absorption of glucose by the inhibition of carbohydrate hydrolyzing enzymes, mainly α-amylase and α-glucosidase, in the digestive organs [4]. Therefore, inhibition of these enzymes can significantly decrease the postprandial hyperglycemia after a mixed carbohydrate diet and can be a key strategy in the control of diabetes mellitus [5].

We have previously reported that Amadori compounds can reduce carbohydrate absorption in the small intestine by inhibiting carbohydrate hydrolyzing enzymes in both *in vitro* and *in vivo* animal model [6]. Korean red ginseng has been shown to have various biological effects, including antioxidant activity, and anti-diabetic, antitumor and stress relieving effects [7–9]. During steaming and drying processes that are necessary for the production of Korean red ginseng, certain components undergo non-enzymatic browning reaction, otherwise known as a Maillard reaction. In the early stage of Maillard reaction, Amadori compounds such as arginyl-fructosyl-glucose (AFG) and arginyl-fructose (AF) are formed through Amadori rearrangement of arginine with glucose or maltose, respectively [10,11].

In our previous study, AF was chemically synthesized and the inhibitory activities against rat intestinal α-glucosidases and porcine pancreatic α-amylase were investigated *in vitro* and in animal model [6]. We reported that dietary AF reduced postprandial glucose level in Sprague-Dawley (SD) rat model via inhibition of carbohydrate hydrolysis enzymes. This was the first report for the potential of AF for type 2 diabetes management. However, there is still limited information in the literature about the dietary effect of long-term AF supplementation on type 2 diabetes management and particularly on hyperglycemia *in vivo*.

Therefore, the aim of this study is to investigate the effect of long-term dietary supplementation of AF on the type 2 diabetes management using *db*/*db* mice, a genetic model for type 2 diabetes. In this study, AF was chemically synthesized and administrated for 42 days in *db*/*db* mice. The effect of long term administration of AF was compared to acarbose and control for fasting glucose levels, HbA1c, total cholesterol and triglyceride contents. Data from the current study provide the potential mechanism of action of AF for the management of type 2 diabetes and also help design future clinical trials.

## 2. Results and Discussion

### db/db Mice Trial

**Figure 1.**
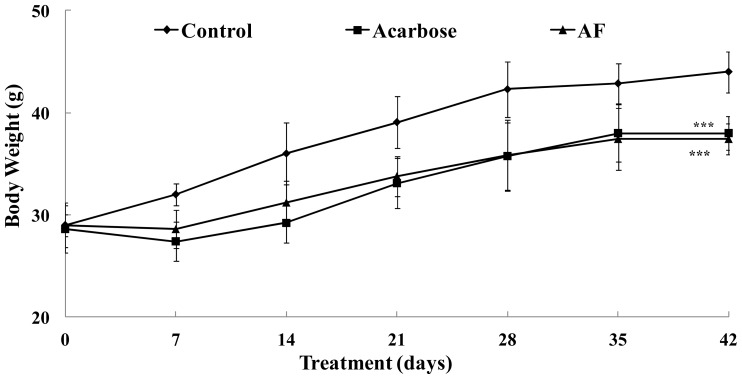
Changes in body weight gains after administration of arginyl-fructose (AF). Male *db*/*db* mice were free access to a high carbohydrate-diet with AF (4%), acarbose (0.04%), and vehicle for 6 weeks. Each point represents mean ± standard deviation (SD). (*n* = 10). Body weight levels were compared between control and treatment groups at each time point by unpaired Student’s *t*-test (*** *p* < 0.001).

**Figure 2.**
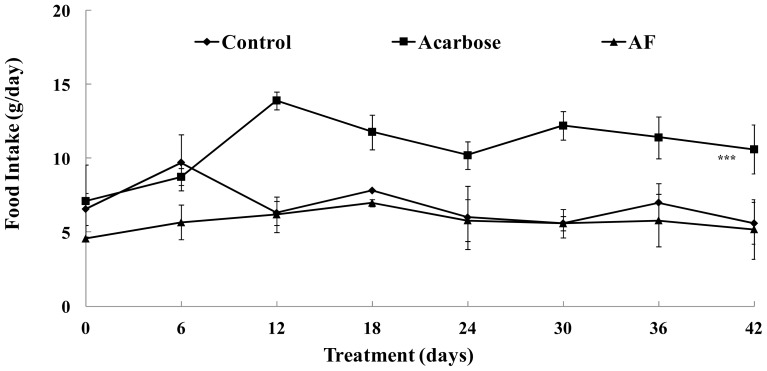
Changes in food intake after administration of AF. Male *db*/*db* mice were free access to a high carbohydrate-diet with AF (4%), acarbose (0.04%) or vehicle for 6 weeks. Each point represents mean ± SD (*n* = 10). Food intake levels were compared between control and treatment groups at each time point by unpaired Student’s *t*-test (*** *p* < 0.001).

The effect of arginyl-fructose (AF) administration was evaluated in *db*/*db* mouse model for 42 days and compared to the effect of acarbose. After 42 days we observed that the body weight of AF treated group was similar to acarbose treatment and significantly lower compared to control (*p* < 0.001) (Figure 1). A significant difference between control and treatments (AF and acarbose) can be identified after 14 days of administration (Figure 1). In addition, we observed that the AF and control animals had similar levels of food consumption, while food intake was dramatically increased in the acarbose group (Figure 2).

Effects of AF treatment after 42 days on fasting blood glucose, HbA1c, HDL-cholesterol, triglyceride content, and cecum weight were also evaluated as shown in Table 1. We observed that fasting glucose level was significantly reduced with AF treatment to the level similar to acarbose (*p* < 0.001) (Table 1). The fasting blood glucose level of control group was around 558.6 (mg/dL), while the levels of AF and acarbose groups were 264.2 and 223.8 (mg/dL), respectively (Table 1). Similarly, the control group had HbA1c levels around 9.8%, while AF and acarbose resulted in significantly lower levels (7.1% and 5.1%, respectively) (Table 1). Triglyceride levels of AF and acarbose were found to be similar amounts (Table 1). HDL-cholesterol was significantly increased only with AF supplementation (157.1 mg/dL), while control and acarbose had similar HDL-cholesterol levels (121.4 and 101.6 mg/dL, respectively) (Table 1). Finally, the cecum weight was determined at the end of the experiment and acarbose treatment group had the largest cecum (1.60 g), followed by control (0.28 g), while AF treatment had the smallest cecum weight (0.27 g) (Table 1).

We observed that the body weight, fasting glucose levels, and HbA1c levels of AF treated group were similar to acarbose treatment and significantly lower compared to control (Figure 1, Table 1). The above findings suggest that AF is preventing the progression of obesity and diabetes due to carbohydrate-rich diet in *db*/*db* mice, in similar manner to the known α-glucosidase inhibitor, acarbose (Figure 1, Table 1), without having the side-effect of excessive α-amylase inhibition observed with the acarbose treatment that results to significantly increased cecum weight (Table 1). The major side effect of acarbose administration is flatulence and diarrhea resulting from the excessive inhibition of starch breakdown. This inhibition of pancreatic α-amylase by acarbose may induce major adverse effects such as abdominal distention, flatulence, meteorism, and diarrhea a consequence of undigested carbohydrates entering the colon where they are used as nutrients for bacterial growth [12,13]. The differences in cecum weight and volume among the control, acarbose, and AF groups are shown in Table 1. Acarbose administration resulted in a 5-fold increase in the weight and volume of the cecum compared with the control and AF, which is consistent with a previous study [12,13]. Based strictly on cecum observations, we can suspect that AF supplementation results to weak inhibition of α-amylase that results to slight increase of cecum weight when compared to the control (Table 1).

Our observations suggest that AF supplementation in *db*/*db* mice along with high starch diet results to fasting blood glucose level, HbA1c, and total weight reductions to a similar level as acarbose, α-glucosidase inhibitor on the market (Figure 1, Table 1). AF and acarbose administration has similar trend and effect on fasting blood glucose levels, HbA1c, and body weight (Figure 1, Table 1). HbA1c is a scientifically advanced test that measures the average blood glucose level over the entire previous 12 weeks period and can accurately evaluate the long-term blood sugar management [14]. Studies have found that HbA1c is an excellent marker of metabolic wellness, with reduced levels being associated with enhanced health. Every unit decrease in HbA1c (e.g., from 6 to 5) has been found to be associated with, for example, significant reductions in heart attacks (−14%), peripheral blood vessel disease (−43%), death due to diabetic complications (−21%), and cataracts (−19%) [14]. Interestingly, the control group had HbA1c levels around 9.8%, while AF and acarbose resulted in significantly lower levels (7.1% and 5.1%, respectively) in this study (Table 1).

## 3. Experimental Section

### 3.1. Materials

Corn starch, casein, vitamin mix, mineral mix, calcium phosphate and sodium chloride were purchased from Raon Bio (Yongin, Korea). Total cholesterol and total triglyceride kits were purchased from Stanbio laboratory (LiquiColor^®^ Test series, Boerne, TX, USA). Blood glucose tester was purchased from Caresens (I-SENS, Anyang, Korea) and HbA1c analyzer was purchase from Infopia Inc. (Clover A1c™, Anyang, Korea). Standard arginyl-fructose was purchased from Proteinworks Co. (Daejeon, Korea). Unless noted, all chemicals were purchased from Sigma-Aldrich Co. (St. Louis, MO, USA).

### 3.2. Animal and Study Design

Five-week-old male C57BL/KsJ-*db*/*db* (*db*/*db*) mice were purchased from Joongang Experimental Animal Co. (Seoul, Korea) and fed a Pico 5053 diet (Oriental Bio. Co., Seongnam, Korea) for 1 week. The animals were housed in individual cages in a room with a 12 h light/dark cycle (lights on from 06:00 h) with 50% ± 7% relative humidity. In this study, ten *db*/*db* mice were used for each group. All mice were adapted to a meal-feeding schedule of free access to Pico 5053 diet with or without samples for 6 weeks (Table 2). The experimental protocols were approved by the Institutional Animal Care and Use Committee (IACUC) of the Hannam University (Approval number: HNU2012-003). The mice had free access to tap water throughout the experimental period. The mice were anesthetized with pentobarbital and killed, and blood was collected. The cecum weight was determined using analytical balance after biopsy.

### 3.3. Blood Analysis

The blood glucose level was measured with a glucose analyzer (CaresensII, I-SENS Inc., Anyang, Korea) using the glucose oxidase method, and the plasma total cholesterol and total glyceride concentration was measured using a kit (Liquicolor^®^ test series, Stanbio Laboratory, Boerne, TX, USA). The concentration of HbA1c was measured using Nycocard reader (Clover A1c™, Infopia Inc., Anyang, Korea).

### 3.4. Statistical Analysis

All data are presented as mean ± SD. Statistical analyses were carried out using the statistical package SPSS 10 (Statistical Package for Social Science, SPSS Inc., Chicago, IL, USA) program and significance of each group was verified with the analysis of One-way ANOVA followed by the Student’s *t*-test for comparison of means.

## 4. Conclusions

One of the therapeutic approaches for decreasing postprandial hyperglycemia is to retard absorption of glucose by the inhibition of carbohydrate hydrolyzing enzymes, α-amylase and α-glucosidase, in the digestive organs [7,8]. Therefore, inhibition of these enzymes can significantly decrease the postprandial hyperglycemia after a mixed carbohydrate diet and can be a key strategy in the control of diabetes mellitus [9–17]. Due to the high enrichment of arginine in crude ginseng, AF is the major Amadori compounds formed by the reaction of maltose, arginine and glucoserespectively during the steaming and heat-drying processes of Korean red ginseng preparation. Compared to the ginsenosides content (0.5%~1.1% in Korean red ginseng) [18,19], commercial red ginseng could have significant levels of AF and AFG, ranging from 0.4% to 2.5% and 0.3% to 2.6%, respectively [6]. Therefore, with the increasing recognition of the various therapeutic effects of Korean red ginseng, application of AF as health food or alternative medicine needs to be thoroughly evaluated. In this manuscript we report that AF can effectively manage the fasting blood glucose and HbA1c levels in *db*/*db* mice, in a similar manner to acarbose. Here, we show in an animal model that the mechanism involves inhibition of carbohydrate hydrolysis enzymes. Our findings provide evidence for the potential application of AF for the management of type 2 diabetes, that need to be further confirmed in a clinical level.

## Figures and Tables

**Table 1.** Effect of AF and acarbose treatment on various parameters in *db*/*db* mice.

Parameters	*db*/*db* mice		

	Control	Acarbose	AF
Glucose (mg/dL)	558.6 ± 57.9	223.8 ± 77.0 ^***^	264.2 ± 21.5 ^***^
HbA1c (%)	9.8 ± 1.0	5.1 ± 0.80 ^***^	7.1 ± 0.6 ^***^
HDL-Cholesterol (mg/dL)	121.4 ± 25.6	101.6 ± 18.8	157.1 ± 4.5 ^**^
Triglyceride (mg/dL)	217.0 ± 22.7	129.2 ± 18.3 ^***^	130.4 ± 26.9 ^***^
Cecum (g)	0.28 ± 0.09	1.60 ± 0.40 ^***^	0.27 ± 0.07

Each point represents mean **±** SD (*n* = 10). All parameter were compared between control and treatment groups at 42 days by unpaired Student’s *t*-test (^**^ *p* < 0.01; and ^***^ *p* < 0.001).

**Table 2.** Composition of diets (g/kg).

High carbohydrate diets	Control	AF	Acarbose
Corn Starch	661	621	660.6
Casein	226	226	226
Soybean oil	60	60	60
Vitamin mix (1)	31	31	31
Mineral mix (2)	9	9	9
Calcium phospahte	10	10	10
Sodium chloride	3	3	3
Sample (AF)	–	40	–
Acarbose	–	–	0.4

(1) Vitamin mixture: AIN-93VX;

(2) Mineral mixture: AIN-93G.
